# Variation between species, populations, groups and individuals in the fitness consequences of out-group conflict

**DOI:** 10.1098/rstb.2021.0148

**Published:** 2022-05-23

**Authors:** Amy Morris-Drake, Patrick Kennedy, Ines Braga Goncalves, Andrew N. Radford

**Affiliations:** School of Biological Sciences, University of Bristol, 24 Tyndall Avenue, Bristol BS8 1TQ, UK

**Keywords:** conspecific rivals, group living, mortality, out-group conflict, reproductive consequences, social conflict

## Abstract

Out-group conflict is rife in the natural world, occurring from primates to ants. Traditionally, research on this aspect of sociality has focused on the interactions between groups and their conspecific rivals, investigating contest function and characteristics, which group members participate and what determines who wins. In recent years, however, there has been increasing interest in the consequences of out-group conflict. In this review, we first set the scene by outlining the fitness consequences that can arise immediately to contest participants, as well as a broader range of delayed, cumulative and third-party effects of out-group conflict on survival and reproductive success. For the majority of the review, we then focus on variation in these fitness consequences of out-group conflict, describing known examples both between species and between populations, groups and individuals of the same species. Throughout, we suggest possible reasons for the variation, provide examples from a diverse array of taxa, and suggest what is needed to advance this burgeoning area of social evolution.

This article is part of the theme issue ‘Intergroup conflict across taxa’.

## 1. Fitness consequences of out-group conflict

In social species across the animal kingdom, groups regularly interact with conspecific outsiders. These interactions can be peaceful or simply involve information exchange [1,2], but often there is conflict if the outsiders are seeking valuable resources such as food, sleeping sites, territory space, matings and breeding positions [3–5]. Threats may come from single individuals, same-sex coalitions or rival groups; we use ‘out-group’ conflict to refer to that with any conspecific outsider(s), and ‘intergroup’ conflict to refer to that with other groups specifically. Traditionally, work on out-group conflict has focused on contest behaviour; for instance, variation in the contributions of different group members, reasons that interactions escalate from signal exchanges to physical fights, and factors influencing who wins [2,3,6–10]. Indeed, a recent systematic review of the topic found that 91% of 394 papers included at least some investigation of contest characteristics [11]. However, there is now also increasing research quantifying behavioural [see 4] and, most importantly, fitness [see 12] consequences of out-group conflict.

The most obvious fitness consequences are those that can arise immediately to participants in a physical contest. For instance, there can be loss of life, extra-group matings, transfer of females between groups and replacement of breeders of both sexes [7,13–17]. However, out-group conflict also generates a much broader range of delayed, third-party and cumulative consequences for survival and reproductive success (reviewed in [12]). For example, a contest could have delayed fitness consequences for participants: individuals injured in fights [18–20] may subsequently have a greater mortality rate and reduced breeding performance [21,22]. Individual contests can also lead to later knock-on consequences for non-participating group members. For instance, a breeding vacancy created by contest-related mortality of the incumbent can be filled by another group member [23]. An outsider taking over a breeding position can generate reproductive opportunities for unrelated opposite-sex individuals [24], but can also cause feticide, infanticide and eviction [25–28]. Moreover, group-size changes can affect mortality risk from predation and starvation, competitiveness in future out-group encounters, offspring survival and the likelihood of group extinction [2,20,29–32].

Beyond contests, some fitness consequences can also arise from interactions with secondary cues of outsiders: for example, close encounters with both rivals or their faecal deposits can lead to disease and parasite transmission [33–35]. More broadly, the general landscape of out-group threat, not just contests, can affect space use, defensive actions (e.g. patrolling and scent-marking), movement, resting, vigilance, and intragroup affiliation and aggression [4,19,32,36–40]. The result could be use of more risky areas, greater energy expenditure, reduced foraging time and lessened parental care [36,41,42]. Finally, there can be cumulative effects of multiple contests or the build-up of outsider pressure over time. Groups may lose part or all their territory to rivals, reducing access to resources crucial for survival and both current and future reproductive success [36,43,44]. Furthermore, the cumulative effect of out-group threat probably generates chronic stress [45]. Chronic stress is associated with reduced body condition, increased susceptibility to disease and predation, and lessened investment and success in reproduction for adults [46–50]. There can also be transgenerational effects for offspring [49,51], potentially through maternal effects [52] or conflict-induced decreases in the quality of parental care [41,53].

**Figure 1.**
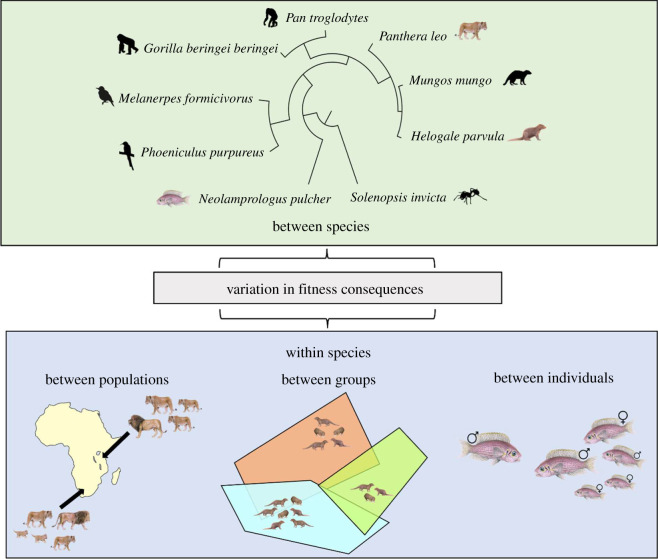
Variation in fitness consequences arising from out-group conflict can occur both between species and between populations, groups and individuals of the same species. Silhouette images from http://phylopic.org. Coloured images from original drawings by Martin Aveling.

The increasing quantification of fitness consequences that can result from out-group conflict makes it timely to consider variation both between and within species. Determining the causes of such variation is important for a full understanding of sociality because out-group conflict is hypothesized to be a powerful selective force in the evolution of, for example, social structure, within-group dynamics, territoriality, cooperation and cognition [54–58]. Interspecific variation in fitness consequences might arise owing to differences in, for instance, dispersal patterns, the composition of groups and inclusive fitness benefits. Intraspecific variation might occur at multiple scales: between populations (e.g. those with different densities or availability of resources), groups in the same population (e.g. those that differ in the number of neighbours or in size) and individuals in the same group (e.g. those of different sex, dominance status or health). More broadly, inter- and intraspecific variation can have far-reaching implications for population dynamics, community structure and ecosystem functioning, including in response to environmental change [59,60]. In this prospective review, we document known variation in the fitness consequences of out-group conflict at a species, population, group and individual level (figure 1). While rather more research has investigated potential reasons for variation between groups and individuals than between species and populations, we suggest possible explanations at all four levels. Throughout, we provide illustrative examples from a wide range of taxa and consider what is needed moving forward to develop our understanding of this widespread, but somewhat neglected, aspect of sociality.

## 2. Variation between species

There exists considerable interspecific variation in the likelihood of mortality for those participating in out-group interactions. In some species, many interactions with neighbouring groups are peaceful (e.g. bonobos, *Pan paniscus* [1]), even sometimes involving a paradoxical lack of discrimination and blurring of identity between in-group and out-group individuals (e.g. unicolonial ants [61]). In species where out-group contests do commonly occur, there is still variation in likely mortality: in some, such as green woodhoopoes, *Phoeniculus purpureus*, contests essentially never escalate to violence, being decided by vocal and visual displays [2]; in others, such as pied babblers, *Turdoides bicolor*, contests can sometimes (*ca* 10% of occurrences) involve physical fighting (e.g. pecking and leg grappling) which can result in injuries but rarely death [62]; while in yet others, including chimpanzees, *Pan troglodytes*, banded mongooses, *Mungos mungo* and some dampwood termites (e.g. *Zootermopsis nevadensis*), contests are often lethal [51,63,64]. For example, intergroup aggression accounted for 17% of adult deaths in one well-studied chimpanzee population [65] and 10% of total adult mortality in banded mongooses [17]. Some of the interspecific variation in mortality is driven by the cause of out-group contests: in chimpanzees, banded mongooses and greater anis, *Crotophaga major*, for instance, coordinated raids are made into rival territories in a targeted attempt to kill adults or offspring [66–68], which increases the likelihood of fatalities. Such coalitionary killing through raiding has been suggested to arise when there is a major imbalance in power (e.g. in species where big differences in group size occur), which is especially true in fission–fusion societies ([69,70], but see [71]). As a direct contrast, groups of vervet monkeys, *Chlorocebus pygerythrus*, prefer to intrude on neighbouring territories when the owners are not in the area, thus avoiding direct conflict and minimizing the mortality risk [72]. The likelihood that contests escalate is also dependent on the benefits of securing the disputed resource and the costs of violence (the risk of injury or death). For instance, access to out-group females may be more valuable for male chimpanzees than bonobos, since the operational sex ratio is more heavily male-skewed (and hence there are fewer within-group mating opportunities) in the former [73]. Not only are there interspecific differences in overall contest-related mortality rates but in who dies in battle: there are sex and dominance-related differences between species that, to at least some extent, mirror differences in contest participation (see *Individual-level variation*).

Species also differ in the extent to which out-group conflict leads to reproductive opportunities. In some, most opportunities to become a new reproductive arise when the existing dominant dies or leaves, so successors are typically groupmates (e.g. *Polistes* wasps [74]). In others, a large proportion of replacements are owing to enforced takeovers, and so successors are outsiders (e.g. geladas, *Theropithecus gelada* [75]). This distinction is not absolute as there are, for example, species where vacancies are filled by out-group individuals: in some stingless bees, for instance, foreign queens regularly take over queenless colonies (e.g. *Melipona scutellaris* [76]); and in acorn woodpeckers, *Melanerpes formicivorus*, rival coalitions of outsiders battle to fill vacant breeding positions [77]. Enforced takeovers might be expected to be more likely in species where same-sex coalitions combine their efforts to usurp current breeders, as is the case in geladas, pied babblers and African lions, *Panthera leo* [15,75,78]. Which sex tends to be replaced by outsiders also differs between species. Female-biased dispersal predominates in birds; for example, in pied babblers and Arabian babblers, *Argya squamiceps*, females are more likely than males to disperse and aggressively takeover breeding positions [79,80]. By contrast, male-biased dispersal predominates in mammals; for instance, takeovers in meerkats, *Suricata suricatta*, are almost always by males [81,82]. There are exceptions to this general taxonomic difference, however, with mating system, relatedness patterns and competition for limiting resources all important in explaining interspecific variation [83]. Reproductive opportunities also arise through sneaky extra-group matings during intergroup interactions (IGIs) [17,84]. The scope for such matings may depend on factors as varied as the amount of vegetation cover and the level of group cohesion during contests, but two may be especially important: how much access there is to unrelated opposite-sex individuals within the group, and the extent to which mate-guarding can be circumvented. These latter two factors are illustrated in a comparison of meerkats (only *ca* 3% of pups are fathered by out-group males [85]) and banded mongooses (18% of pups are sired by out-group males [86]). Breeding female meerkats typically have access to an unrelated in-group male, and so have little incentive to pursue out-group matings, and meerkat groups are despotic, meaning that most females do not breed and thus mate-guarding is relatively easy for the dominant male. By contrast, banded mongoose females are more likely to be related to the males in their own group and groups are more egalitarian, so multiple females come into oestrus simultaneously and mate-guarding them all is thus challenging.

Out-group conflict may be a major avenue of parasite and disease transmission in some species—for example, rabies transmission by biting in out-group confrontations in red foxes, *Vulpes vulpes* [87]—but not in others, such as birds where contests are predominantly decided through displays [6]. Infection can arise not only from physical contact with rival groups or rovers but from inspection of scent-marks (e.g. tuberculosis in meerkats and banded mongooses, respectively [33,35]). The risk of infection might therefore be expected to be higher in mammals, where inspection of scent-marks is common, relative to birds. As with the transmission of non-pathogenic microbes [88], pathogen transmission can occur between groups via three main pathways: territory and resource overlap, out-group aggression and temporary between-group movements and dispersal events. Species that have overlapping territories or resource use—neighbouring groups of dwarf mongooses, *Helogale parvula*, sometimes use the same sleeping burrow on consecutive nights, for instance [89]—probably come across secondary cues of rivals, or indeed parasites left from prior occupancy, more often than those who do not share physical spaces. This risk can be minimized if groups avoid areas within their home ranges that have recently been used by neighbouring groups, as is the case in mountain gorillas, *Gorilla beringei beringei* [90]. Out-group conflict and parasite transmission could be further connected by positive feedbacks: if groups are weakened by parasites, they may be targeted by rivals exploiting the imbalance of power, who then contract the parasite in turn (e.g. the spread of *Varroa* mites in honeybees, *Apis mellifera* [91]).

The influence of out-group conflict on within-group social behaviour [4] and probable associated fitness consequences—receipt of affiliation or aggression may lead to short-term hygiene and mating benefits [2,92,93] and cumulative, long-term changes in an individual's status and hence reproductive prospects [94]—seems to differ between species. In some, there are changes to behaviour during contests [10,95], whereas in others the changes occur in the aftermath of an IGI [37,38,96,97]. There are also differences in whether affiliative and/or aggressive behaviour is affected, as well as the direction of the behavioural change. For example, increases in post-IGI affiliation are seen in green woodhoopoes, dwarf mongooses and mountain gorillas [37,38,96], but a decrease was documented in banded mongooses and Javan gibbons, *Hylobates moloch* [98,99]. At least some of the interspecific variation in within-group behavioural interactions during contests is probably generated by differences in kinship structure, patterns of philopatry and the shareability of resources [100], as well as the extent to which the inclusive fitness interests of group members are aligned in competing against a rival group. In principle, members of fission–fusion societies may have less alignment of interests than cooperative breeders; and in advanced eusocial groups, there is near complete alignment of interest [101]. In species where individuals have stronger temptations to defect (e.g. leave the group, seek extra-group matings or fail to contribute to the contest), threats or rewards may be more necessary [10,16,95]. Increases in within-group affiliation in the aftermath of out-group interactions are often interpreted from a functional perspective to increase ‘social cohesion’ [38,96,102,103], although that general term may obscure a diversity of phenomena: for example, honest signalling of willingness to collaborate rather than to compete; sharing of information about threat levels; pre-emptively coordinating the group for future contests; rewarding participation; strengthening dyadic bonds or coalitions through the exchange of services; and reconfirming or testing dominance hierarchies. Species vary in the importance of the different forms of ‘social cohesion’; for instance, signalling cooperativeness is only needed in those without an intrinsic common interest in cooperation.

Finally, the cumulative effect of out-group conflict can potentially have consequences for reproductive success but, again, there is variation in those documented for the limited number of species studied. For example, while there is no effect of neighbour pressure on reproductive success in Tasmanian native hens, *Tribonyx mortierii* [104], it is negatively correlated with chimpanzee inter-birth intervals, and neighbour pressure during pregnancy is also associated with lower offspring survival post-birth [49]. By contrast, intergroup conflict in crested macaques, *Macaca nigra*, is associated with high foetal survival and no changes to infant survival [105]; similarly, banded mongoose intergroup conflict is associated with higher foetal survival, possibly owing to reduced intragroup sexual conflict in response to infanticide [51]. Some caution is needed when interpreting these results as they arise (understandably) from long-term correlative datasets; a recent experimental test with the tractable cichlid fish species *Neolamprologus pulcher* found a negative effect of chronic out-group conflict on reproductive success [106]. One possible driver of interspecific variation in reproductive consequences concerns the food on which different species are reliant. If foraging is focused on scarce, locally clumped, monopolizable resources (e.g. ripe fruit for chimpanzees), losing groups probably face higher nutritional stress and, hence, potentially lower foetal survival and quality. By contrast, where species rely more on dispersed, abundant, small food items (e.g. the invertebrate prey of banded mongooses), the loss of intergroup contests might have less or no impact on early reproduction. Out-group pressure might manifest in reproductive consequences through chronic stress [107,108] and/or disruption to within-group social relationships [109], and there may also be lasting instability following breeding takeovers, but much future work is needed to unpick these possibilities.

While it is increasingly clear that species can differ vastly in the fitness consequences of out-group conflict, current explanations (including our own) are often *post hoc* suggestions relating to idiosyncratic life-history or ecological differences. There is a strong need for theoretical modelling to generate testable predictions; this approach has demonstrably aided our understanding of other aspects of out-group conflict, such as the factors influencing individual participation in contests and the determinants of group success [110–112]. Phylogenetically controlled meta-analyses can then be used to test those predictions and to uncover underlying correlates of interspecific variation (e.g. [113]). However, to be robust, such meta-analyses need data from a much greater number of species; currently, relatively few detailed studies have quantified out-group impacts on, for instance, disease transmission, parental care and reproductive output. Ideally, clear metrics of out-group conflict would be established and consistently used in studies; these might be individual measures of, for example, the landscape of out-group pressure, frequency of out-group interactions and contest intensity, or some combined index [49,58]. Often, relevant data need to be accumulated over many generations and/or years, which is one of several reasons why long-term studies tracking known individuals are so valuable [114].

## 3. Variation within species

Variation in fitness consequences arising from out-group conflict can occur at several levels within the same species: between populations, groups and individuals. At each level, much of the variation arises from differences in the frequency, intensity and outcome of interactions between conspecific rivals (table 1). For instance, an increased frequency of out-group interactions will, all else being equal, result in more time and energy invested in conflict, a greater risk of injury or disease transmission, and longer-lasting, cumulative consequences for survival and reproductive success. Interactions that are resolved after initial visual or vocal signalling probably carry fewer costs than those that escalate to physical fighting; and interactions that last longer not only carry greater costs in terms of time and energy invested but might result in subsequent reductions in contributions to cooperative behaviours such as offspring care [41,99]. Losing a contest probably results in more negative consequences than winning, though winning does not necessarily preclude costs [18], and there can be considerable variation in the type and magnitude of the consequences, as well as which individuals suffer these. Elucidating reasons for variation in out-group interaction frequency, intensity and outcome are therefore crucial, as well as establishing more nuanced differences in the fitness consequences.

**Table 1.** Potential differences at the population, group and individual level that could explain intraspecific variation in the frequency and intensity of out-group interactions and involvement in them, as well as factors that affect the type and magnitude of the consequences in the aftermath of a contest (its outcome).

	populations	groups	individuals
frequency	population density; territory spacing; pathogen pressure; seasonality; anthropogenic effects	number of neighbours; presence of valuable resources; breeding vacancies; changing group size	personal threat; personal costs; reward and punishment; involvement of kin or strongly bonded groupmates
intensity	inter- and intragroup relatedness; breeding system	relative resource-holding potential; rival identity; resource value asymmetry; interaction location	personal threat; personal costs; reward and punishment; involvement of kin or strongly bonded groupmates
outcome	resource availability; disease risk; predation risk; habitat degradation	winning versus losing; rival identity	resource lost; change in group members

### (a) Population-level variation

Different populations of the same species may vary in the likelihood of out-group conflict, and therefore in the magnitude of consequences, for a range of reasons. First, demographic factors can amplify or diminish the value of competing with outsiders. For instance, in the Ngorongoro Crater, Tanzania, which has a high population density of African lions, females are more likely to respond to experimentally simulated territory intrusions than do lionesses in the Serengeti, Tanzania, which has a lower population density [115]. Similarly, trematode colonies within snails may invest in a bigger ‘standing army’ in areas with higher prevalence of competitors [116]. Diana monkey, *Cercopithecus diana*, groups in a forest with high population density (where intergroup competition is high) exhibit greater aggression towards neighbours cf. strangers (‘nasty neighbour’ behaviour), while groups in a low-density forest exhibit lower aggression towards neighbours cf. unfamiliar rivals (‘dear-enemy’ behaviour) [117]. Second, spatial factors can drive variation in the frequency of out-group interactions. For instance, green woodhoopoe groups inhabiting linear riverine valleys in South Africa face challenges at only a maximum of two, relatively narrow, territory margins [2], while those from a Kenyan population that inhabits open woodland are often surrounded by neighbours on all sides and thus experience threats from multiple directions [118]. Chacma baboons, *Papio ursinus*, restricted to movement up and down desert canyons are less likely to detect intrusions than troops densely packed on open swamps [119]. Third, latitudinal gradients in pathogen pressure may drive differences in antagonism towards out-groups [120]. Where pathogen prevalence is higher, infection can be reduced by avoiding contact with other groups, which could influence IGI rates. As a final general example, anthropogenic effects can intensify animal out-group competition through habitat loss or the introduction of novel resources. For instance, in banded mongoose populations living near human habitation, groups may coalesce at food-waste sites, increasing the likelihood of intergroup encounters [121].

The frequency of IGIs in a population may also vary seasonally, especially if key contested resources (e.g. water, food and mating opportunities) are more or less plentiful at different times of the year, with implications for territory size, group overlap and mortality [122,123]. For example, some studies find that IGIs are more frequent in the breeding season [51,124], while others find the opposite pattern [125]. Seasonal differences might be because territories are only defended for part of the year, when the relevant resources (e.g. nesting or mating sites) are required [126]. In those species that do defend year-round territories, seasonal variation in defensive activities and IGIs is commonly argued to be adaptive: increases in the breeding season may, for instance, be owing to the benefits of increased defence or information gathering about potential competitors or mates at that time [127,128]. Moreover, there could be season-specific behaviours that increase the likelihood of IGIs: for example, banded mongoose groups are most likely to be involved in a contest when females are in oestrus compared to any other period [51] because those individuals initiate intergroup contests to sneak matings with out-group males [17]. However, seasonal differences in the behaviour of permanent territory-holders might also be the consequence of variation in food availability, which is typically lower in the non-breeding season [129]. In pied babblers, for instance, there are fewer IGIs during the non-breeding season compared to the breeding season, but this is in part owing to differences in food availability; supplementary feeding in the non-breeding season when foraging success was lower led to an increase in responsiveness to the simulated intrusion of a rival group [124]. In general, population differences in diet and food availability are likely to influence key aspects of sociality, including group formation and structure, as well as the occurrence of IGIs [55,130–132].

Populations may vary not only in the frequency of out-group conflict but also in both the type and magnitude of consequences arising from it. For instance, there are population differences in the occurrence of lethal violence in chimpanzees, at least partially owing to variation in the extent to which additional group members lend their support once an IGI has started [133]. Moreover, whereas male chimpanzees from the population in Taï National Park, Côte d'Ivoire kidnap females without being violent towards these ‘prisoners’, males in Gombe National Park, Tanzania subject females to severe aggression which is probably lethal in some cases [73,133]. It is theoretically possible that demographic differences between populations may also impact the indirect fitness consequences of conflict, by altering intra- and intergroup relatedness. Moreover, losing an out-group contest (or sustaining an injury from contest participation) may be more costly in a population with limited or fluctuating resources, high disease risk, higher predation or habitat degradation, compared to in populations inhabiting less-challenging locations.

Moving forward, we need to understand what drives between-population variation in both levels of out-group conflict and the associated fitness consequences; most previous studies have focused on one or the other (and usually the former). An unusually detailed picture of both aspects has been worked out in red fire ants, *Solenopsis invicta* [134,135]. Populations are either monogyne (where each colony has only a single queen) or polygyne (where each colony has multiple queens). Monogyne populations experience intense intercolony conflict, but polygyne populations show an absence of intercolony conflict (supercoloniality) and frequently share workers. This variation can be traced directly to a ‘social chromosome’ supergene (a cluster of tightly linked loci): *BB* homozygotes at the *Gp-9* locus are monogyne, whereas *Bb* heterozygotes are polygyne (with the *b* allele acting as a greenbeard allele whose workers reject any *BB* homozygote queens). The variation in intercolony conflict also has demonstrable fitness consequences: compared to polygyne populations, colonies in monogyne populations defend territories against other colonies, which greatly reduces intercolony contact and thus lowers the transmission levels of a severe queen-killing pathogen. Even in this excellent example, with strong evidence for the proximate mechanism identified, more still needs to be learned: for instance, what factors sustain the polymorphism and its functional significance.

### (b) Group-level variation

Within a population, each group experiences a particular level of outsider pressure over a given timeframe, leading to variation in the consequences of out-group conflict. For instance, groups probably have different numbers of neighbours and differ in their spatial positioning relative to others [136,137]. In general, a group with more neighbours and/or a more central territory has a greater likelihood of an IGI arising than a group with fewer neighbours or that is located on the edge of a population, although this may be complicated by dear-enemy effects [5]. Other factors that could lead to intrapopulation variation in the rate of IGIs include whether: a territory contains a particularly valuable resource, such as a fruiting tree or a female in oestrus, that might attract rival groups or roving males, respectively [14,138]; a group has infants, which rivals might target [139]; there is a breeding vacancy available in a group, which might attract multiple outsiders [77]; or a neighbouring group is changing in size [51]. In banded mongooses, for instance, groups that were growing had more IGIs than groups that were shrinking, possibly owing to the need to forage over larger areas and, therefore, expand beyond their existing territorial boundaries [51]. However, it is also possible that groups shrinking in size could exert high levels of pressure if they are looking to increase their group size via kidnapping, as seen in pied babblers [140]. An increased frequency of IGIs not only enhances the likelihood of short-term consequences but also cumulative effects on reproductive output [49].

The consequences of out-group conflict can also vary for the same group between contests in which it is involved depending on, for example, the intensity (i.e. duration and level of escalation) of each one. For many species, longer and escalated contests are more likely when the resource-holding potential (RHP) of the interacting parties is similar, as it may take more time to assess the competitive ability of the rival and require physical aggression to reach an outcome [2,141,142]. In black howler monkeys, *Alouatta pigra*, for example, groups that contain an equal number of males have longer IGIs than those where there is an asymmetry [141]. However, in some species this effect of RHP similarity is not necessarily apparent: in tufted capuchins, *Sapajus apella*, for instance, neither males nor females showed a decreased probability of approach when the numerical odds strongly favoured the opposing group [143]. Here, resource context appears more important [143]; as another example of this type of variation, grey-cheeked mangabey, *Lophocebus albigena*, groups that had recently arrived at a location were more likely to approach playback of rivals than those who had been there longer, with site residency probably indicating the degree of short-term, local resource exploitation [144]. Rival identity is also known to affect the intensity of IGIs: for instance, mountain gorillas exhibit greater tolerance towards groups containing familiar or related individuals [142,145]; in pied babblers, conflicts with kin groups are shorter than those with non-kin rivals [62]. Moreover, for species that exhibit dear-enemy or nasty-neighbour relationships, interactions with groups representing the greater threat might be expected to be more intense [5]. Lastly, contest location is a key factor that can affect its intensity. Contests at the core of a territory, which is generally considered more valuable owing to plentiful resources [146,147], can be more intense than those in peripheral areas, as seen in mountain gorillas, black and white colobus, *Colobus guereza* and blue monkeys, *Cercopithecus mitis* [130,145,148].

The fitness consequences of out-group contests will also differ depending on the outcome, with losing having negative effects both immediately through loss of the disputed resource [44,149] and in the aftermath of the interaction. For instance, behavioural or space-use changes are often more extreme after losing [36,96,150]. In addition, the consequences of losing could vary depending on the identity of the rival. For example, in green woodhoopoes, strangers are seeking to take over the whole territory while neighbours only invade temporarily [151], making a loss to the former of greater lasting consequence. While winning a contest can, by contrast, translate into positive consequences through the acquisition of the disputed resource [64,139], a recent study of acacia ants, *Crematogaster mimosa*, showed that winning groups can also incur important costs. Colonies that won physical contests still suffered a reduced workforce (owing to mortality during fighting), which resulted in compromised defence against predators and neighbouring conspecifics in the future [18]. It is often differences in RHP that determine the outcome of intergroup contests. For some species, especially those in which most group members contribute to contests, asymmetries in total group size are a key deciding factor [6,20,148]. In other cases, where only a subset of a group contributes (e.g. just males), group composition can be a better predictor of outcome than group size [152,153]. In grey wolves, *Canis lupus*, for example, groups are more likely to win if they have a greater number of older individuals or adult males participating, even if they are the smaller group [154]; in wedge-capped capuchins, *Cebus olivaceus*, and tufted capuchins, relative male group size is the most important predictor of IGI outcome [155,156]; whereas in black and white colobus, groups with fewer but larger adult males are more likely to win IGIs [152]. These differences in RHP between groups, sometimes modified by additional factors such as contest location [136,157], therefore, contribute to group-level variation in fitness consequences.

By contrast to species- and population-level variation in the fitness consequences of out-group conflict, more studies have explicitly investigated reasons for group-level variation. This is probably owing to the relative ease of collating data from different groups in the same population as opposed to at a broader scale, especially in terms of using consistent definitions and methods. Much of the focus on group-level variation has been on differences in contest intensity and outcome; moving forward, we still need to consider in greater depth what influences outsider pressure level (see [49] for calculation of a multifaceted outside pressure index). For instance, while a group dominance hierarchy exists among neighbours in some species, including African lions, grey wolves, Verreaux's sifaka, *Propithecus verreauxi* and black and white colobus [44,154,158,159], we are only starting to understand how this may impact the level of out-group pressure experienced and its influence on variation in fitness consequences across groups [125]. In addition, it is becoming increasingly apparent that nuanced relationships can exist between neighbouring groups owing to other factors such as relatedness and familiarity [145]; it is important to capture these within the ‘intergroup dominance hypothesis' [160]. Information on the relative strengths of different groups potentially also extends beyond near neighbours. For example, in acorn woodpeckers, individuals from territories up to 3 km away travel to witness power struggles taking place for breeding vacancies before returning to their own territory; such spectators are trading off the gathering of social information against the risk to their home territory created by their absence [77]. Finally, there are members of the population without territories that should be incorporated into this broader consideration of outside pressure levels: there are floating individuals [161], roving males travelling between groups during the breeding season [14] or splinter groups trying to establish a territory [162]. Taking this overarching view and studying the complex network of out-group conflict will help to shed light on how the pressure exerted by outsiders varies between groups and how that translates into differences in fitness consequences.

### (c) Individual-level variation

Differences in out-group contest participation can lead to variation in fitness consequences among group members, both immediately and with a delay. Intuitively, the more involved an individual is in a physical contest, the greater the risk to health and life, as evidenced in banded mongooses and red fire ants [17,163]. This risk level will depend, to at least some extent, on the intensity of a given interaction (see above). Intriguingly, there can be variation in which group members instigate interactions with rivals—for instance, females in vervet monkeys [10] and grey-cheeked mangabeys [164], while males in chimpanzees [73]—but the instigators do not necessarily suffer the greatest costs [17]. Variation in participation may also have knock-on impacts for within-group behaviour [4]. For instance, during prolonged contests between neighbouring groups, female vervet monkeys are affiliative towards participating male groupmates, as reward for current contribution and plausibly to foster continued participation, and aggressively punish uncooperative males [10]. Similarly, there is evidence from a variety of species that individuals participating more in an out-group contest receive increased affiliation in the aftermath [96,102]. These changes in within-group interactions may have short-term benefits with respect to, for instance, hygiene and stress levels [2,93]. Longer term benefits may arise from improved social network position and the strength of social bonds with groupmates, which are known to influence, for example, reproductive success and life expectancy [94,165]. Contest participation might also result in immediate reproductive benefits if, for example, males are rewarded with matings by females [92,166].

Individual variation in out-group contest participation occurs both between species and contests. At a species level (see also *Variation Between Species*), there are social invertebrates that have evolved specialized warrior castes (e.g. turtle ants, *Cephalotes rohweri* [167]; trematode spp. [116]); these individuals overwhelmingly deal with conspecific competition and thus bear the costs. In vertebrates, sex and dominance most commonly affect participation levels. All group members might contribute to some extent but to different degrees [41,84]; there are species in which one or other sex predominantly contribute—males in species as varied as Tasmanian native hens [104], grey wolves [168], tufted capuchins [132] and bonnet macaques, *Macaca radiata* [169], while greater female participation occurs in blue monkeys [170]. Dominant individuals often contribute more than subordinate groupmates, either because they have priority access to the disputed resources [171,172] or have more at stake [173], but subordinates may contribute more when they suffer higher costs than dominants from a lost contest (e.g. owing to heightened food competition with immigrants [6]). Variation in participation can also differ from contest to contest depending on a variety of factors. These include intruder identity and thus the threat presented (see *Population-level variation* and *Group-level variation*), as well as a range of individual factors related to reproduction, health status or the behaviour of groupmates. For instance, pregnancy and the presence of dependent young have been shown to decrease female participation levels in vervet monkeys and common marmosets, *Callithrix jacchus* [84,171], but to increase them in mountain gorillas and Verreaux's sifakas [172,174], and to have no effect in black howler monkeys [175]. Good body condition may reduce the costs of fighting [176], while poor health status may impact territoriality—for instance, space use in wood mice, *Apodemus sylvaticus* [177], travelling distances in grey wolves [178] and territory size in Tasmanian devils, *Sarcophilus harrisii* [179]—and thus alter the likelihood and quality of participation in out-group encounters. Finally, the involvement of kin and/or groupmates to whom an individual is strongly bonded can potentially enhance the likelihood of contest participation, as seen in chimpanzees [180].

Regardless of participation, the outcome of out-group conflicts can have distinct consequences for different group members. Contests that result in the partial loss of territory or access to shelter may affect all individuals similarly [3,36]. By contrast, the loss of access to particularly valuable but limited food resources probably impacts females and dependent young more than other group members, given the importance of food resources for female reproductive success and offspring growth [181], while the loss of a mating opportunity to a rover affects the cuckolded individual the most [14,182]. Arguably, the most extreme inter-individual differences in fitness consequences arise following breeder replacements by outsiders, as evidenced by African lions following a pride takeover by out-group males. Usurped males lose future reproductive success through the loss of breeder status, and may lose current reproductive success through infanticide and eviction or killing of older offspring by the incoming males; replaced males may also suffer severe injury or even death during contests [26,78]. In addition to the almost inevitable loss of dependent young, reproductively active females may experience temporary infertility following takeovers [78]. For other female group members, however, there may be an increase in reproductive success through access to unrelated males as daughters rarely mate with members of their father's coalition [183], while the new breeder benefits the most, having acquired multiple females with whom to breed [78].

Several aspects of individual variation in participation and in the subsequent fitness consequences of out-group conflict remain to be explored in detail. First, although the disparity of outcomes to different group members from a single interaction has been highlighted in several systems (e.g. lions, vervet monkeys, banded mongooses; see above), less consideration has been given to how single individuals may both gain and lose from a single interaction with rivals. For instance, individuals may gain or maintain territory but lose a partner or be injured; time spent interacting with rivals may have secondary impacts on the individual's offspring owing to a reduced level of care or increased vulnerability to predators [41,184]. Second, while changes to within-group interactions in the aftermath of out-group conflict have been proposed to induce greater group cohesion, there has been limited consideration of how these behavioural changes may link to future contest participation; increases in within-group affiliation and aggression during contests with rivals can affect participation levels [9,10,95], but we know little about the influence on contests arising days or weeks later. Third, a little-considered source of variation is the presence of conspecifics not directly involved in the conflict (i.e. third parties). Audience effects are known to influence contest behaviour in dyadic interactions between single individuals [185,186] but eavesdropping and its consequences in relation to out-group conflict have received little research attention [187]. The presence of other group members as an audience might be important: for instance, male white-faced capuchins, *Cebus capucinus*, responded more strongly to playback of an out-group male when tested in the presence of a male groupmate than when tested alone [188], although this might be owing to the potential support available rather than an audience effect *per se*. Moreover, because many group-living species defend territories whose borders are shared with multiple neighbour groups [25,90], and individuals from further afield may come to spectate on contests between rival coalitions [77], outsider audiences are a potentially important source of variation in out-group behaviour and thus its consequences.

## 4. Conclusion

Out-group conflict is probably a potent evolutionary force in species across the animal kingdom [54–58], so quantifying the fitness consequences and determining the reasons for interspecific and intraspecific variation can greatly enhance our understanding of sociality. Our aim with this review has, therefore, been twofold. First, to showcase some, at least, of what is already known about the extensive variation in fitness consequences between species, populations, groups and individuals. Second, because currently we are often only speculating about the drivers of those differences, to stimulate further empirical and theoretical research by pinpointing some outstanding questions at each level of variation. Moving forwards, effort should be made on standardizing metrics relating to out-group conflict that can be used across taxa, to facilitate direct comparisons between studies. Tests of variation between species and populations, in particular, will probably benefit from collaboration among different research groups; certainly, the sharing of (unpublished) data accumulated gradually from long-term studies will allow otherwise impossible comparative analyses. We advocate that, where possible, future studies combine investigation of both out-group and intragroup conflict, as fitness consequences are probably a result of their interaction but not necessarily in a simple or easily predicted way [49,58]. Moreover, we see value in cross-fertilization between work on humans and non-human animals; research on other organisms can provide valuable insight into our own evolution as the management and impacts of intergroup conflict are a core component of human history ([189,190], but see [191]). Finally, there is a need to consider how environmental change influences out-group conflict and its consequences—climate warming has been suggested to increase the likelihood of conflict, for example [192]—especially given the unprecedented rate at which human activities are altering both terrestrial and aquatic landscapes. As research into the fitness consequences of out-group conflict continues to gain momentum, there will be an increasing appreciation of its importance in shaping social evolution.

## Data Availability

This article has no additional data.
